# Nurse-led pulmonary rehabilitation: a cornerstone for healthy aging in chronic respiratory diseases

**DOI:** 10.3389/fpubh.2026.1827982

**Published:** 2026-04-23

**Authors:** Xue-li Zhang, Si Qin, Jun Jiang, Hong Huang, Yan-ping Cao, Yan-li Yang

**Affiliations:** 1 Department of Respiratory and Critical Care Medicine, The First Hospital of Jilin University, Changchun, China; 2 Department of Orthopedics, The Third Affiliated Hospital of Changchun University of Chinese Medicine, Changchun, China; 3 Jilin Province Electric Power Hospital, Changchun, China

**Keywords:** chronic respiratory diseases, healthy aging, multimorbidity, nurse-led pulmonary rehabilitation, patient-centered care, self-management

## Abstract

The global aging population has driven a substantial increase in chronic respiratory disease (CRD) prevalence, profoundly threatening functional independence and quality of life among older adults. Although pulmonary rehabilitation constitutes a grade A evidence-based intervention, traditional center-based models encounter persistent implementation challenges, including inadequate referral rates, limited accessibility, and poor patient adherence. This perspective article argues that nurse-led pulmonary rehabilitation (NLPR), distinguished by its holistic, continuous, and patient-centered approach, represents not merely an alternative service delivery model but a fundamental strategy for operationalizing healthy aging principles within CRD management. By integrating multimorbidity management, catalyzing self-efficacy, and bridging hospital-community care transitions, NLPR reshapes health trajectories for older adults living with respiratory conditions. This perspective article elaborates four pillars through which NLPR contributes to healthy aging: physiological restructuring, psychological safeguarding, social participation fostering, and advance care planning integration. It further examines implementation barriers, including policy gaps and workforce development needs, while identifying research priorities spanning health economics, protocol personalization, and outcome measurement expansion. Ultimately, NLPR offers a transformative pathway for reconceptualizing CRD from inevitably disabling conditions toward manageable states compatible with meaningful living in later years.

## 1 Introduction

### 1.1 Epidemiological landscape

The accelerating global aging population has significantly intensified the burden of chronic respiratory diseases (CRD), particularly chronic obstructive pulmonary disease (COPD) and interstitial lung disease (ILD) (1). These conditions demonstrate disproportionately high prevalence and disability rates among older adults. COPD, the third leading cause of death worldwide, affects over 10% of individuals aged 60 years and older (2). Meanwhile, the incidence of ILD, most notably idiopathic pulmonary fibrosis, rises exponentially with advancing age (3). These diseases progressively and irreversibly impair lung function while diminishing quality of life through recurrent acute exacerbations, reduced exercise tolerance, and complex multimorbidity (4). The resulting direct medical expenditures and indirect social care costs impose substantial and sustained strain on global public health systems (5).

Although COPD and ILD are both categorized under the umbrella of chronic respiratory diseases, they represent distinct clinical entities with fundamentally different pathophysiological mechanisms and disease trajectories (6). COPD is primarily characterized by persistent airflow limitation associated with chronic airway inflammation, whereas ILD comprises a heterogeneous group of disorders marked by progressive pulmonary fibrosis and restrictive ventilatory impairment (7, 8). This heterogeneity has important implications for clinical management, particularly in the context of pulmonary rehabilitation, where disease-specific adaptations may be required (9, 10). In this perspective, the inclusion of both conditions is intended to reflect the shared challenges faced by aging populations—especially functional decline, reduced exercise capacity, and the high prevalence of multimorbidity—rather than to imply a uniform therapeutic approach.

In addition to these clinical differences, disparities in the evidence base further complicate the application of pulmonary rehabilitation across CRD subtypes (10). Compared with the extensive body of research in COPD, evidence supporting pulmonary rehabilitation in ILD remains relatively limited and less standardized (10, 11). Emerging studies suggest that pulmonary rehabilitation can improve exercise tolerance and health-related quality of life in ILD populations (9); however, important uncertainties persist regarding optimal program structure, long-term sustainability of benefits, and the need for disease-specific tailoring of interventions (11–13). This imbalance highlights a critical gap in current knowledge and underscores the necessity for further research to establish evidence-based, phenotype-adapted rehabilitation strategies for diverse CRD populations (10, 14).

### 1.2 Redefining healthy aging in the context of CRD

Within this context, the conventional paradigm of disease-free survival proves increasingly inadequate. For the substantial population living with CRD, achieving healthy aging requires shifting focus from reversing organic pathology toward preserving optimal functional capacity despite chronic illness (15). This perspective redefines successful aging: maintaining independence in daily activities, preserving cognitive function, and sustaining social participation (16). The objective is to prolong periods of life lived with autonomy and dignity, even as underlying disease progresses. Thus, the critical intersection of geriatric and respiratory medicine lies in identifying interventions that enable older patients to “live well with disease,” reconciling extended longevity with preserved quality of life (17, 18).

### 1.3 Pulmonary rehabilitation paradox

Pulmonary rehabilitation represents a cornerstone, grade A evidence-based intervention for CRD patients, consistently demonstrating efficacy in alleviating symptoms, enhancing exercise capacity, and improving health-related quality of life (10). However, a striking paradox emerges between this robust evidence base and persistently low rates of patient participation and program completion (19). The limitations inherent in traditional, center-based pulmonary rehabilitation models—typically specialist-directed and facility-dependent—substantially contribute to this disconnect. Such models remain inherently resource-intensive, constrained by requirements for specialized facilities, equipment, and multidisciplinary personnel, which fundamentally limits their accessibility (19). Moreover, older patients face considerable logistical barriers, including travel burdens and transportation difficulties, compounded by inadequate transitional support following acute exacerbations (20). These factors frequently precipitate refusal or premature discontinuation. This substantial gap between pulmonary rehabilitation’s proven efficacy and its real-world effectiveness underscores the urgent necessity to develop and validate novel, decentralized rehabilitation strategies better aligned with the circumstances and needs of the aging population.

## 2 Unique value of nurse-led models: expanding the boundaries of traditional pulmonary rehabilitation

### 2.1 Paradigm shift from disease treatment to holistic care

Nurse-led models fundamentally reconceptualize pulmonary rehabilitation by shifting the focus from disease-centered interventions toward comprehensive, patient-centered care (10). This paradigm shift holds particular significance for older adults with CRD, who typically present with complex multimorbidity encompassing cardiovascular conditions, osteoporosis, anxiety and depression, and sarcopenia (21). Nurses, by virtue of their holistic training and practice orientation, are uniquely positioned to function as integrators of multimorbidity management (22). Within pulmonary rehabilitation programs, nurses can seamlessly coordinate pharmacological management, nutritional support, and psychological interventions alongside respiratory-specific components, thereby addressing the interconnected dimensions of patient health that traditional siloed approaches often neglect (10). Furthermore, nurses serve as astute assessors of functional status throughout the rehabilitation continuum. Their sustained engagement in daily patient care enables continuous monitoring of activities of daily living, facilitating timely identification of functional decline or improvement (23, 24). This ongoing assessment capacity allows for dynamic adjustment of rehabilitation goals in response to patients’ evolving capabilities, aligning precisely with the functional paradigm central to healthy aging (25). Rather than treating functional metrics as static baseline measurements, nurse-led approaches recognize function as a fluid outcome requiring responsive intervention.

### 2.2 Empowerment theory and catalyzing self-management

The educational dimension of nurse-led pulmonary rehabilitation (NLPR) derives substantial advantage from the therapeutic relationships nurses cultivate with patients (22). Through sustained clinical contact characterized by continuity and trust, nurses establish foundations for effective health communication that physician-centered models, constrained by time limitations, rarely achieve (22). This relational foundation enables deployment of evidence-based communication strategies, including teach-back methods, to ensure patients properly acquire and retain essential self-management competencies (26, 27). These competencies encompass correct inhaler technique, systematic symptom monitoring, and energy conservation principles critical for maintaining functional independence despite physiological limitations (26). Beyond knowledge transmission, nurse-led approaches leverage motivational interviewing techniques to catalyze sustained behavioral transformation (28). The challenge confronting pulmonary rehabilitation extends beyond initial patient engagement to encompass long-term adherence to prescribed exercise regimens following program completion (29). Nurses, through their accessibility and ongoing patient contact, can provide the sustained motivational support necessary for patients to internalize rehabilitation principles and integrate them into enduring lifestyle modifications (30, 31). This transformative process converts pulmonary rehabilitation from a time-limited intervention into a sustainable foundation for ongoing self-management.

### 2.3 Bridging continuity of care

**Figure 1:**
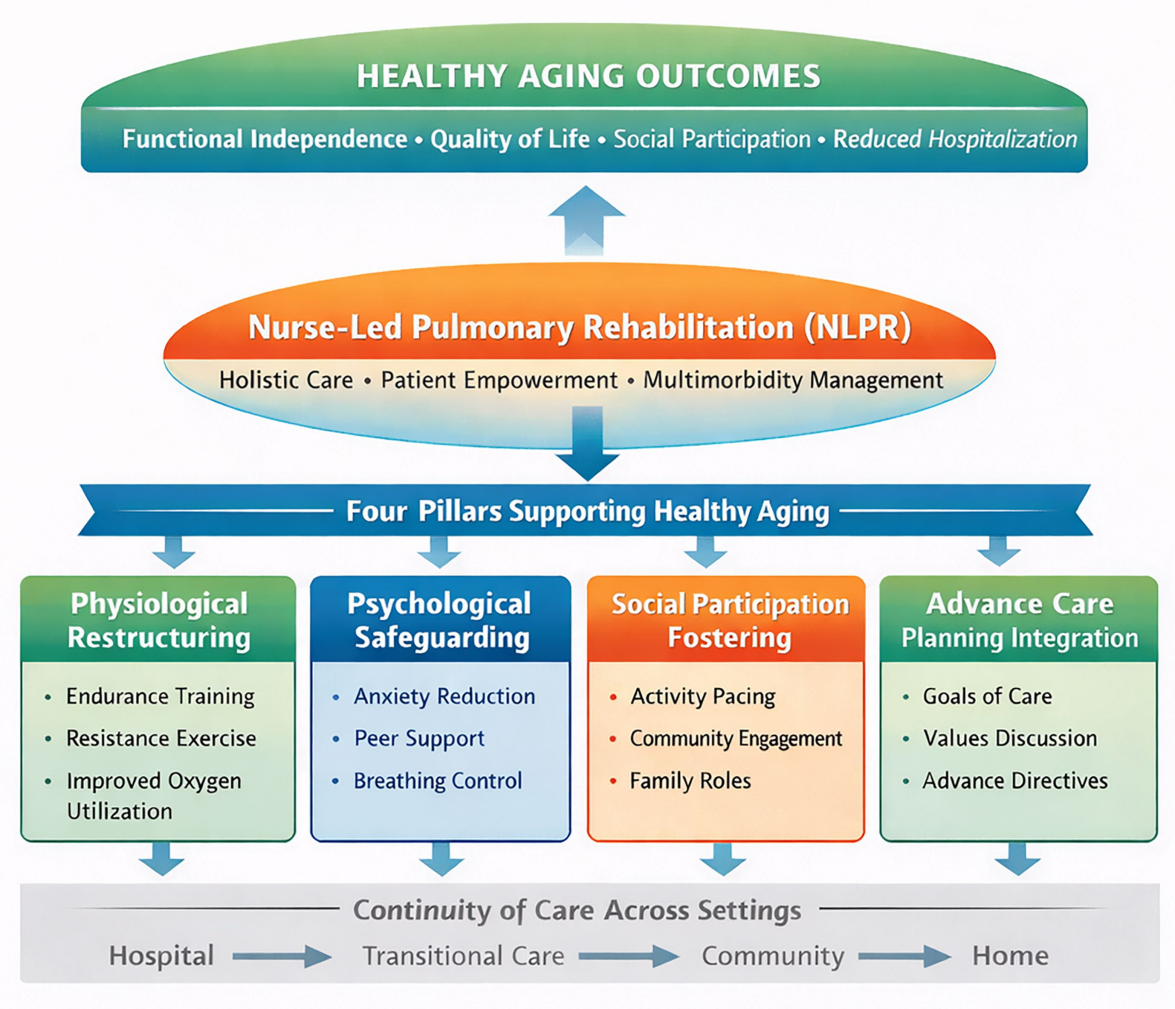
Conceptual framework of nurse-led pulmonary rehabilitation promoting healthy aging in chronic respiratory diseases.

Perhaps most critically, nurse-led models address the fundamental discontinuity characterizing traditional pulmonary rehabilitation delivery. Conventional center-based programs typically operate within acute care settings, creating substantial barriers for patients transitioning between hospital, community, and home environments (27). Nurses function as essential bridges across these care transitions, particularly during the vulnerable post-discharge period following acute exacerbations (32). Through structured transitional care visits or home-based rehabilitation programs, nurses ensure pulmonary rehabilitation continues seamlessly across the hospital-community-home continuum (33). This continuity function prevents the deleterious cycle of functional decline frequently observed when rehabilitation is interrupted during care transitions (32). By maintaining consistent therapeutic engagement regardless of patients’ location within the healthcare system, nurse-led models sustain the momentum of functional improvement and prevent the setbacks associated with care fragmentation (10). For older adults whose functional reserves are already compromised by CRD, this continuity represents not merely convenience but essential protection against irreversible decline (34). The bridging function of NLPR thus operationalizes the principle that effective rehabilitation cannot be confined to institutional settings but should accompany patients throughout their healthcare journey. The conceptual framework through which NLPR promotes healthy aging is illustrated in Figure 1.

## 3 Pathways to healthy aging: four pillars of NLPR

This perspective article delineates four core pillars by which NLPR advances healthy aging. These domains are designed to address the multifactorial needs of older adults with CRD in a holistic manner. The core interventions, underlying mechanisms, and expected clinical contributions of these four pillars are summarized in Table 1.

**Table 1:** Four pillars of nurse-led pulmonary rehabilitation and their clinical contributions.

Pillar	Core interventions	Mechanism	Expected outcomes
Physiological restructuring	Endurance training, resistance exercise	Improve oxygen utilization, reduce sarcopenia	Improved exercise capacity, preserved independence
Psychological safeguarding	Breathing training, peer support, psychological counseling	Reduce dyspnea-related anxiety, improve coping	Reduced depression/anxiety, enhanced resilience
Social participation	Activity pacing guidance, community engagement	Restore ability to participate in family and social activities	Improved social connectivity and life satisfaction
Advance care planning	ACP discussions, values clarification	Align medical care with patient preferences	Improved end-of-life care quality

ACP, advance care planning.

### 3.1 Restructuring physiological function

NLPR establishes the restructuring of physiological function as its foundational pillar through individualized, progressive exercise training (10). This approach combines endurance and resistance components tailored to the capabilities of older adults with CRD (10). Endurance training, typically involving walking or stationary cycling, improves cardiorespiratory fitness by enhancing oxygen utilization efficiency (10). Resistance training, using elastic bands or light weights, addresses sarcopenia, the age-related muscle loss that disproportionately affects respiratory patients (35). The synergistic effect of combined training enables NLPR to delay or partially reverse functional decline, thereby preserving patients’ capacity for ambulation and self-care activities (19). These physiological improvements translate directly into maintained independence, representing the most fundamental contribution of pulmonary rehabilitation to healthy aging outcomes. By systematically rebuilding physiological reserve, NLPR provides the biological foundation upon which all higher-order functional capabilities depend.

### 3.2 Safeguarding psychological wellbeing

The second pillar addresses the psychological dimensions of living with CRD, which profoundly influence both quality of life and rehabilitation outcomes. Older adults with CRD frequently experience disease-specific fears, particularly dyspnea-related anxiety that triggers activity avoidance and subsequent functional decline (36, 37). Social isolation and depression commonly accompany these conditions, compounded by reduced mobility and diminished social engagement opportunities (37). NLPR incorporates structured psychological support mechanisms to address these challenges. Group-based rehabilitation sessions, delivered in person or through digital platforms, facilitate peer support networks that normalize disease experiences and reduce isolation (38, 39). Additionally, NLPR integrates breathing techniques derived from mind–body practices such as yoga and tai chi, adapted for respiratory patients (40, 41). These techniques serve dual purposes: improving respiratory efficiency while reducing anxiety through enhanced parasympathetic activation. The resulting improvement in psychological resilience enables patients to approach their condition with reduced fear and greater confidence in their self-management capabilities.

### 3.3 Fostering social participation

The third pillar extends rehabilitation outcomes beyond physiological and psychological metrics to encompass meaningful social engagement. NLPR explicitly recognizes that healthy aging requires not merely functional capacity but its application within social contexts (42). As exercise training progressively enhances activity tolerance, patients regain capability to participate in family responsibilities and community activities previously abandoned due to respiratory limitations (43). This renewed participation capacity requires structured guidance on activity pacing and energy conservation, enabling patients to sustain engagement without precipitating exacerbations (44, 45). The social connections fostered through this process represent a higher-order rehabilitation outcome, contributing to the sense of purpose and belonging central to quality of life in later years (10, 19). By explicitly targeting social participation as a rehabilitation goal, NLPR distinguishes itself from models focused exclusively on physiological improvement and aligns fully with the multidimensional conceptualization of healthy aging (46).

### 3.4 Integrating advance care planning

The fourth pillar addresses the often-neglected domain of end-of-life preparation within chronic disease management frameworks. Nurses, by virtue of their sustained proximity to patients throughout the rehabilitation journey, occupy an optimal position to facilitate advance care planning discussions (47). Unlike acute care settings where such conversations occur under crisis conditions, NLPR provides a longitudinal context within which patients can gradually explore and articulate their values, goals, and preferences regarding future care (48). Nurses can integrate these discussions naturally into ongoing rehabilitation contacts, framing them as components of comprehensive self-management rather than as separate, threatening conversations (49). This approach ensures that advance care planning occurs before acute deterioration, when patients retain decisional capacity and can communicate preferences authentically (50). The resulting advance directives, informed by patients’ lived experience with chronic disease, enable care alignment with personal values during future health crises. This integration of advance care planning within NLPR ensures that healthy aging encompasses not only the quality of living but also the dignity of dying, completing the continuum of person-centered respiratory care (51).

## 4 Challenges and future directions

### 4.1 Current implementation barriers

The widespread adoption of NLPR faces several systematic barriers that require resolution before established evidence can translate into routine clinical practice. Policy and reimbursement frameworks represent a primary obstacle, as many healthcare systems lack specific billing codes for NLPR services (52). Without dedicated reimbursement mechanisms, healthcare institutions struggle to sustain NLPR programs financially, relegating them to pilot status or grant-dependent operations rather than integrated services (19). This policy gap reflects broader challenges in valuing preventive and rehabilitative interventions within healthcare systems oriented primarily toward acute care reimbursement.

Workforce development constitutes another critical barrier, as NLPR requires specialized competencies beyond standard nursing education. The absence of standardized training programs and certification pathways for NLPR practitioners limits the availability of qualified personnel (10, 53). Effective NLPR delivery demands that nurses possess competencies in exercise prescription, comprehensive assessment of multimorbidity, and adaptation of rehabilitation protocols for patients with complex health profiles (10). Establishing accredited training curricula and certification standards represents an essential prerequisite for workforce capacity building.

Technology integration presents both opportunities and implementation challenges. Remote NLPR delivery through digital applications and wearable monitoring devices offers potential solutions for geographic accessibility barriers, particularly for patients in rural or underserved areas (54). However, realizing this potential requires addressing digital literacy disparities among older populations, ensuring device affordability, and developing evidence-based protocols for remote supervision that maintain safety and efficacy comparable to center-based programs (55).

### 4.2 Future research priorities

Advancing the NLPR evidence base requires prioritized investigation across several domains.

An important foundational gap lies in the limited evidence addressing older adults with multimorbidity, who represent the predominant population in real-world CRD settings (56, 57). Current pulmonary rehabilitation trials frequently exclude patients with complex comorbid profiles, thereby constraining the external validity and clinical applicability of their findings (58). Future research should therefore prioritize pragmatic study designs that more accurately reflect real-world clinical complexity, incorporating common coexisting conditions such as cardiovascular disease, frailty, cognitive impairment, and polypharmacy. Addressing this gap is essential for ensuring that NLPR models are both generalizable and adaptable to routine clinical practice (59).

Health economic evaluation stands as a pressing research need, with rigorous randomized controlled trials required to demonstrate NLPR’s cost-effectiveness (60). Such studies should quantify outcomes including hospital readmission reduction, acute exacerbation prevention, and long-term healthcare expenditure savings (61). Demonstrating economic value is essential for justifying policy changes and reimbursement expansion, particularly within resource-constrained settings.

Personalization of rehabilitation protocols represents a second research priority. The heterogeneity of older adults with CRD necessitates investigation into optimal program components based on individual characteristics (19). Research should examine how rehabilitation dosing—including intensity, frequency, and duration—should be stratified according to geriatric syndromes such as frailty and cognitive impairment (62). Understanding these moderating factors will enable transition from one-size-fits-all approaches to precision rehabilitation tailored to patient profiles.

Outcome measurement expansion constitutes a third research imperative. Traditional pulmonary rehabilitation trials have prioritized physiological metrics, particularly lung function parameters and six-minute walk distance (10). Future investigations should broaden outcome assessment to encompass patient-reported measures aligned with healthy aging conceptualizations. Social participation, life satisfaction, and meaningful activity engagement represent outcomes that capture rehabilitation’s contributions beyond physiological improvement. Incorporating these person-centered endpoints into trial designs ensures that NLPR evaluation reflects the multidimensional benefits most valued by older adults themselves.

### 4.3 Controversies and evidence gaps in NLPR

Despite growing recognition of NLPR, several controversies and evidence gaps persist. First, the heterogeneity of CRD populations raises uncertainty about the generalizability of nurse-led models across different disease phenotypes, particularly between COPD and ILD (10). Second, although the short-term benefits of pulmonary rehabilitation are well established, long-term adherence to and sustainability of nurse-led interventions remain insufficiently understood (44). Third, key parameters of NLPR—such as optimal intensity, duration, and delivery mode (center-based, home-based, or digital)—have yet to be standardized (19). Moreover, evidence supporting NLPR in frail older adults with multimorbidity is limited, as these individuals are often underrepresented in clinical trials (62). Finally, economic evaluations and cost-effectiveness analyses specifically targeting NLPR are scarce, which hinders policy implementation (63). Addressing these gaps is essential to advance NLPR from a promising model to a scalable, evidence-based standard of care.

## 5 Summary

NLPR represents far more than a pragmatic response to healthcare resource constraints. It embodies a fundamental alignment with the philosophical principles underlying healthy aging. By shifting focus from disease-centered intervention toward patient empowerment, care integration, and functional preservation, NLPR offers a transformative approach to CRD management. This model reconceptualizes CRD from inevitably disabling conditions to manageable states compatible with meaningful living.

The four pillars elaborated throughout this perspective article demonstrate how NLPR operationalizes healthy aging principles across physiological, psychological, social, and existential domains. Through individualized exercise training, psychological support mechanisms, social participation facilitation, and advance care planning integration, NLPR addresses the multidimensional needs of older adults that traditional disease-focused models systematically neglect. This comprehensive approach recognizes that optimal outcomes extend beyond physiological metrics to encompass the capabilities and experiences that patients themselves value most deeply.

Realizing the full potential of NLPR requires coordinated action across multiple stakeholder groups. Policymakers should establish appropriate reimbursement frameworks that recognize NLPR as an essential service rather than an optional adjunct to respiratory care. Educational institutions should develop standardized training curricula and certification pathways to build workforce capacity. Clinical administrators should prioritize NLPR integration within geriatric and respiratory service delivery structures. Only through such multisectoral collaboration can NLPR transition from evidence-based innovation to embedded standard of care.

## Data Availability

The original contributions presented in the study are included in the article/supplementary material, further inquiries can be directed to the corresponding author.
